# Surgical treatment of traumatic frontal hematoma: comparison of the endoscopic supraorbital approach with frontotemporal approach

**DOI:** 10.3389/fneur.2023.1234009

**Published:** 2023-08-17

**Authors:** Qiang Yang, Min Cui, WeiMing Xiong, YangLingXi Wang, Yang Liu, WeiDuo Zhou, Peng Chen, XiaoYong Tang

**Affiliations:** Department of Neurosurgery, Chongqing Emergency Medical Center, Chongqing University Central Hospital, Chongqing, China

**Keywords:** traumatic frontal hematoma, the endoscopic, the supraorbital approach, the frontotemporal approach, minimally invasive technology

## Abstract

**Background:**

The objective of this study was to compare the efficacy, safety, and outcomes of the endoscopic supraorbital approach and frontotemporal approach for the treatment of traumatic frontal hematoma, with the aim of demonstrating the feasibility of the endoscopic supraorbital approach.

**Methods:**

A total of 24 cases underwent hematoma evacuation, including 10 cases using the endoscopic supraorbital approach and 14 cases using the frontotemporal approach. Baseline demographic data, hematoma clearance rate, blood loss, postoperative complications, and 6-month outcomes were retrospectively analyzed.

**Results:**

Both approaches effectively evacuated the hematoma, with hematoma clearance rates of 90.97 ± 10.23% in the endoscopic supraorbital group and 85.29 ± 16.15% in the frontotemporal approach group (*p* > 0.05). The supraorbital approach group demonstrated significantly shorter operation times compared to the frontotemporal approach group (116.50 ± 28.19 min vs. 193.29 ± 72.55 min, *p* < 0.05), as well as significantly less blood loss (55.00 ± 33.08 mL vs. 685.71 ± 840.20 mL, *p* < 0.05). There was no significant difference in the rate of postoperative complications between the two groups, and the majority of patients achieved favorable outcomes with a Glasgow Outcome Scale score of 4 or 5 in both groups.

**Conclusion:**

Compared to the frontotemporal approach, the endoscopic supraorbital approach offers advantages such as shorter operation times, reduced blood loss, similar treatment effects, and comparable complication rates. Therefore, the endoscopic supraorbital approach may serve as a viable alternative for the treatment of traumatic frontal hematoma.

## Introduction

Traumatic frontal hematoma is a prevalent form of traumatic brain injury in clinical practice, primarily resulting from deceleration injury. In cases where patients exhibit severe clinical symptoms, significant hematoma volume, and evident mass effect, surgical intervention may prove beneficial in improving prognosis (1). Among the surgical techniques employed, frontotemporal craniotomy is a well-established procedure known for its ability to effectively evacuate damaged brain tissue and intracranial hematoma, and achieve decompressive craniectomy. Nevertheless, this method also has obvious disadvantages, including prolonged operation time, extensive tissue damage, and increased blood loss (2).

The supraorbital approach, utilizing the eyebrow arch as the incision, represents a minimally invasive surgical technique that allows access to the anterior and middle cranial fossa through the prefrontal floor channel. This approach is frequently employed for the treatment of conditions such as frontotemporal tumors and anterior circulation aneurysms. Notably, it offers distinct advantages, including shortened operation time, minimal tissue damage, and negligible impact on the patient’s appearance (3). Furthermore, the utilization of endoscopy offers the advantage of providing a wider and clearer surgical view, effectively addressing the limitations associated with insufficient visibility and frequent adjustment of viewing angles during deep operations when compared with traditional microscopy.

Despite the potential merits of the endoscopic-assisted removal of traumatic frontal hematoma via the supraorbital approach, there is a paucity of reports in the existing literature. Moreover, no studies have directly compared the treatment outcomes, safety, and efficacy of this approach with traditional frontotemporal surgery. In the present study, we aim to compare the endoscopic supraorbital approach with the frontotemporal approach for the treatment of traumatic frontal hematoma. Specifically, we will evaluate the operation time, postoperative complications, and overall outcomes between the two groups, with the intended purpose of assessing the feasibility and safety of the supraorbital approach.

## Materials and methods

This study received approval from the hospital review board and was conducted between 2020 and June 2022. A retrospective analysis was performed on a total of 24 patients with traumatic frontal hemorrhage who underwent surgical treatment at our hospital. Among them, 10 patients underwent the endoscopic supraorbital approach, while 14 patients underwent the frontotemporal approach. Informed consent for surgical treatment was obtained from all patients or their family members prior to surgery.

Upon admission, all patients underwent dynamic follow-up with head computed tomography (CT) scans to determine the presence of active bleeding. Surgical treatment was recommended for patients exhibiting progressive loss of consciousness, decreased Glasgow Coma Scale (GCS) score, and significant mass effect due to large frontal hematoma, as indicated by CT examination. Patients with primary brain stem injury, abnormal coagulation function, and vital organ failure were excluded from this study. Patients who underwent decompressive craniectomy were also excluded. The selection of the surgical approach was based on the surgeon’s preference.

For the endoscopic supraorbital approach, the patient was placed in a supine position with the head secured using a head frame, slightly tilted to the opposite side of the lesion. A skin incision of approximately 3–4 cm was made inside and lateral to the eyebrow, which could be extended laterally as necessary. Subcutaneous tissue was carefully dissected, followed by longitudinal incision and separation of the frontalis muscle to minimize injury to the supraorbital nerve and muscle. After reaching the skull, a hole was drilled above the eyebrows, and a bone window measuring 1.5 cm × 2 cm was created. The dura was then incised in an arc with the base toward the orbit. Subsequently, a tubular brain dilator was inserted along with a rigid endoscope (HOPKINS II 0°, STORZ, Germany). Once inside the hematoma cavity, the hematoma was gradually removed, and careful observation of the interface with the brain tissue was performed to achieve maximum hematoma removal. Large hematomas were removed in small pieces. Hemostasis was thoroughly achieved, and the wound was covered with hemostatic gauze. The dura was tightly closed, and the bone was returned without the placement of a drainage tube.

**Figure 1:**
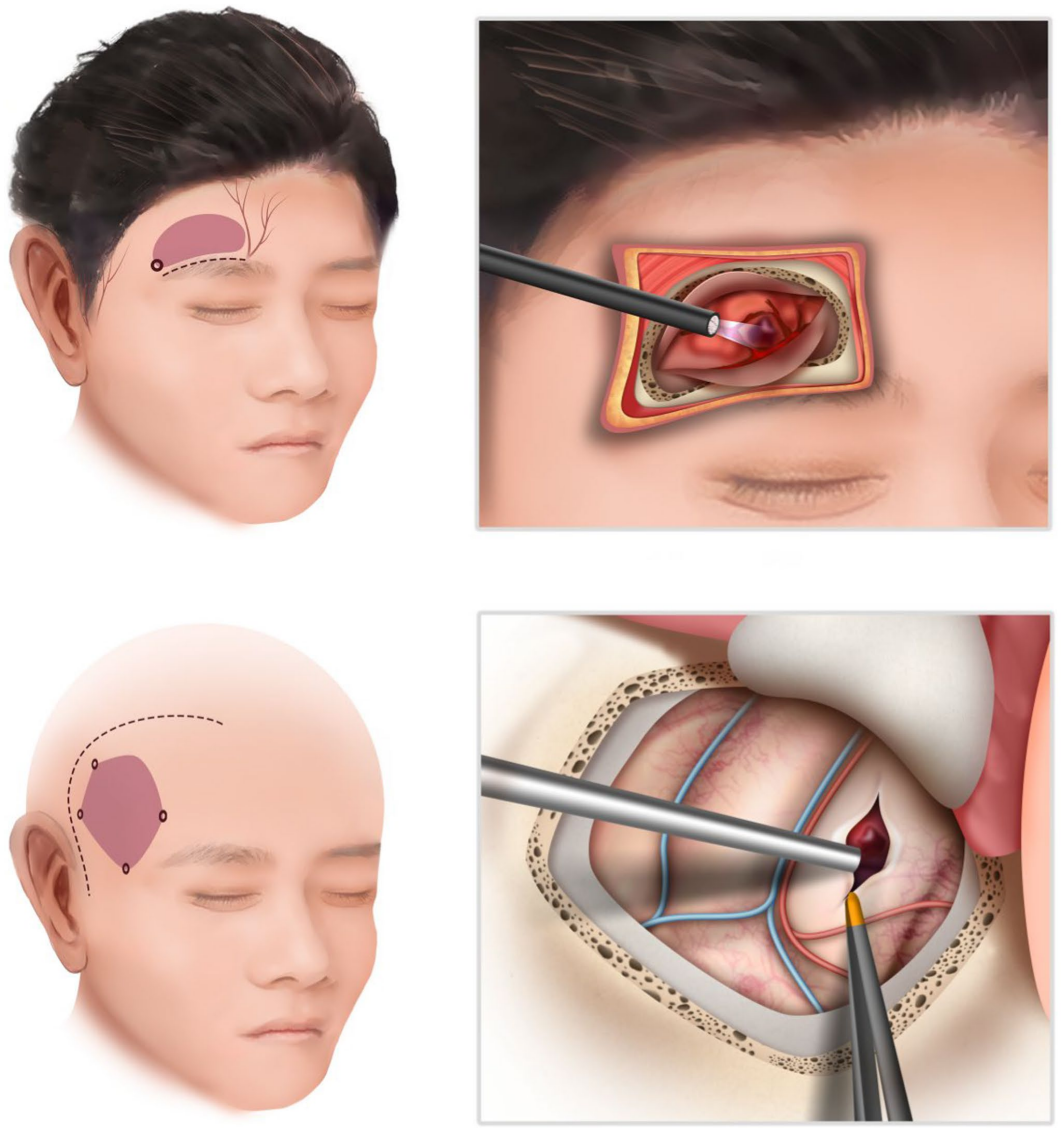
The schematic illustration of the supraorbital approach (above) and the frontotemporal approach (below). The endoscopic supraorbital technique enables a reduction in both the size of the skin incision and the craniotomy.

For the frontotemporal approach, the patient was placed in a supine position, and a conventional method was employed on the side of bleeding. After scalp incision, the skull was drilled to create a bone window measuring 4 cm × 6 cm. The dura was incised and suspended, followed by hematoma removal under microscope guidance. Strict hemostasis was achieved, and routine closure of the skull was performed after dura suturing (Figure 1).

Patient data collected included gender, age, injury factors, hematoma size, GCS scores before the operation, operative time, blood loss, hematoma clearance rate, incidence of rebleeding, and other complications (intracranial infection, ischemia, epidural/subdural hematoma, cerebrospinal fluid leakage, etc.), length of hospital stay, and Glasgow Outcome Scale (GOS) scores at 6 months were also recorded. Hematoma volume under CT scan was calculated using the formula: volume = (length × width × height)/2, and hematoma clearance rate was determined as (preoperative hematoma volume – postoperative hematoma volume)/preoperative hematoma volume × 100%. The GOS score categories were as follows: 1 indicated death, 2 indicated a vegetative state, 3 indicated severe disability, 4 indicated moderate disability, and 5 indicated mild or no disability.

### Statistical analysis

Statistical analysis was performed using SPSS version 20.0 (IBM Corp). Continuous variables were reported as mean ± standard deviation, while categorical variables were presented as *n* (%). T-tests were used to assess relationships between continuous variables, while chi-square or Fisher’s analyses were used for categorical variables. A significance level of *p* < 0.05 was considered statistically significant.

## Results

### The general situation

Demographic analysis of the two groups revealed that the proportion of female patients in the supraorbital group was 20.00% (2/10), while in the frontotemporal approach group it was 28.57% (4/14). The mean age of patients in the supraorbital group was 53.10 years (31–78 years), and in the frontotemporal group, it was 58.43 years (42–77 years). The main causes of injury were falling and traffic accidents, followed by falls from a height (Table 1).

**Table 1:** Demographic characteristics of patients who underwent the endoscopic supraorbital approach and the frontotemporal approach.

Observation index	Endoscopic supraorbital approach (*n* = 10)	Frontotemporal approach (*n* = 14)	*p* value
Sex			*p* > 0.05
Male	8	10	
Female	2	4	
Age (y)	53.10	58.43	*p* > 0.05
Causes of injury			*p* < 0.05
Falling	7	3	
Traffic accident	3	6	
Falling from a height	0	5	
GCS before operation			*p* > 0.05
13–15	3	1	
9–12	6	10	
3–8	1	3	

### Analysis of the surgical effect

**Figure 2:**
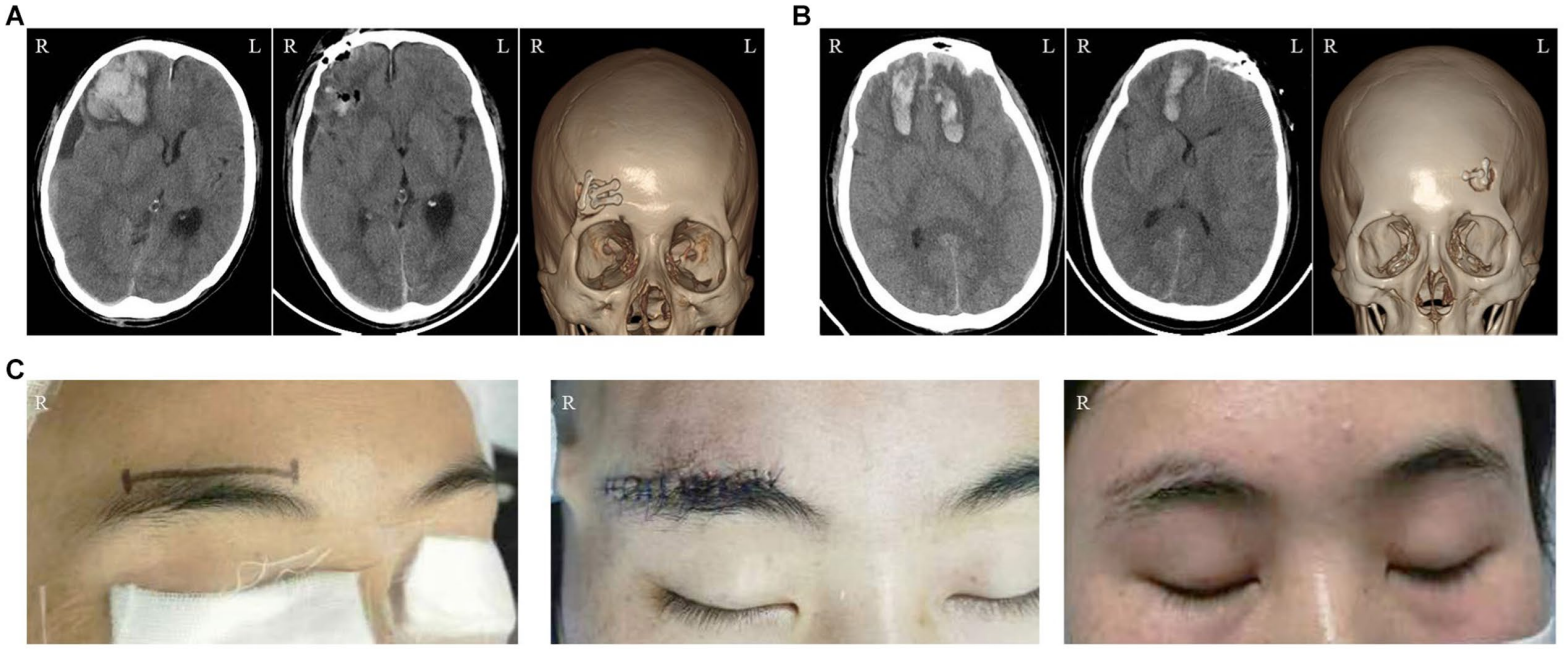
Illustrations of the Endoscopic Supraorbital Approach: **(A)** The imaging data of a patient who suffered from semicomatose consciousness as a result of a traffic accident. The sequence from left to right represents the preoperative CT scanning, postoperative CT scanning, and postoperative CT three-dimensional reconstruction, respectively. **(B)** The imaging data of another patient who underwent the same approach. **(C)** The changes in the appearance of a female patient. The images, from left to right, illustrate the surgical incision, the wound condition after the operation, and the appearance at the 2-month follow-up, respectively.

**Figure 3:**
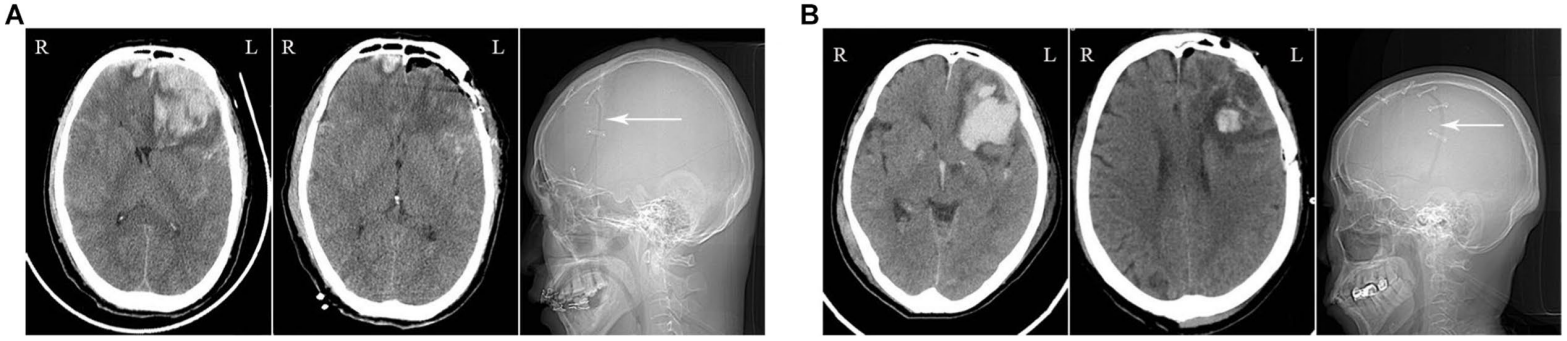
Illustrations of the Frontotemporal Approach: **(A)** The imaging data of a patient who sustained a high fall injury. The preoperative CT scan reveals a large hematoma in the left frontal lobe with mass effect (left image). The postoperative CT scan shows the successful clearance of the hematoma (middle image), with visualization of the craniotomy in the frontotemporal skull (right image, white arrow). **(B)** The imaging data of a patient who suffered from a traffic accident injury.

The preoperative hematoma volume in the endoscopic supraorbital group and frontotemporal group were 34.82 ± 10.80 mL (20.00–60.00 mL) and 34.56 ± 7.55 mL (23.5–48.4 mL), respectively. The average hematoma clearance rate in the supraorbital approach group and the frontotemporal approach group was 90.97 ± 10.23% and 85.29 ± 16.15% (*p* > 0.05), respectively. In the supraorbital approach group, 9 out of 10 patients had a hematoma clearance rate greater than 80%, while in the frontotemporal approach group, 10 out of 14 patients had a hematoma clearance rate greater than 80%. The supraorbital approach group had significantly shorter operation time (116.50 ± 28.19 min) compared to the frontotemporal approach group (193.29 ± 72.55 min, *p* < 0.05). Furthermore, the supraorbital approach group had smaller blood loss (55.00 ± 33.08 mL) compared to the frontotemporal approach group (685.71 ± 840.20 mL, *p* < 0.05). Postoperative follow-up CT scans revealed that one case (10.00%) in the supraorbital approach group experienced rebleeding in the operative area, while the rebleeding rate in the frontotemporal approach group was two cases (14.26%, *p* > 0.05). These patients did not undergo secondary surgical treatment and improved with conservative measures such as hemostasis and dehydration. In the supraorbital approach group, there was one case of intracranial infection (10%) and one case of ischemia (10%) as postoperative complications. In the frontotemporal approach group, one case of intracranial infection (7.14%) and three cases of ischemia (21.34%) were observed, and these complications improved with symptomatic treatment. No cerebrospinal fluid leakage or epidural/subdural hematoma was found in either group. In the frontotemporal group, two patients died from severe injuries. The average length of hospital stay in the supraorbital approach group was similar to that in the frontotemporal approach group (38.80 ± 23.73 days *VS* 45.07 ± 31.43 days, *p* > 0.05). At the 6-month follow-up, most of the patients had a good outcome with a GOS score of 4 or 5 in both groups (Figures 2, 3; Table 2).

**Table 2:** Comparison of perioperative parameters between the endoscopic supraorbital approach group and the frontotemporal approach group.

Observation Index	Endoscopic supraorbital approach (*n* = 10)	Frontotemporal approach (*n* = 14)	*p* value
Hematoma volume before operation (ml)	34.82 ± 10.80	34.56 ± 7.55	*p* > 0.05
Hematoma volume after operation (ml)	3.90 ± 6.03	5.82 ± 7.29	*p* > 0.05
Hematoma removal rate	90.97 ± 10.23%	85.29 ± 16.15%	*p* > 0.05
Operative time (min)	116.50 ± 28.19	193.29 ± 72.55	*p* < 0.05
Blood loss (ml)	55.00 ± 33.08	685.71 ± 840.20	*p* < 0.05
Re-bleeding	1(10.00%)	2(14.26%)	*p* > 0.05
Epidural/Subdural hematomar	0	0	
Intracranial infection	1(10.00%)	1(7.14%)	*p* > 0.05
Ischemia	1(10.00%)	3(21.43%)	*p* > 0.05
Cerebrospinal fluid leakage	0	0	
Length of hospital(days)	38.80 ± 23.73	45.07 ± 31.43	*p* > 0.05
GOS ≥ 4 after 6 months	9	11	*p* > 0.05

## Discussion

Frontal injury is primarily caused by deceleration trauma, resulting in the rubbing of frontal brain tissue against the uneven bone at the base of the anterior cranial fossa. This leads to brain tissue fracture at the bottom of the frontal lobe, vascular injury, and the formation of intracerebral hematoma. Due to the proximity of the hematoma to the cerebral falx, central herniation can easily occur, resulting in consciousness disorders (4). Guidelines suggest surgical intervention for frontal brain injuries with a GCS score of 6–8, hematoma volume exceeding 20cm^3^ with midline displacement exceeding 5 mm, or hematoma volume surpassing 50cm^3^ (5). Research indicates that early surgical intervention may yield better outcomes compared to conservative or delayed surgical approaches. The frontotemporal approach is a conventional surgical method employed for treating frontal lobe injuries. It effectively evacuates damaged brain tissue and hematomas, reducing the risk of secondary injury. Frontotemporal decompressive craniectomy is widely used internationally as it effectively reduces intracranial pressure and prevents cerebral herniation (6). In cases where hematoma removal alone suffices to reduce intracranial pressure, the supraorbital approach is a candidate option.

The supraorbital keyhole approach, an advancement in minimally invasive technology, utilizes the eyebrow arch as the incision point to access the anterior and middle cranial fossa through the prefrontal floor channel. It is commonly used for the treatment of anterior circulation aneurysms and tumors located in the anterior cranial fossa floor, such as tuberculum sellae and olfactory sulcus meningiomas (7, 8). The term “minimally invasive” not only refers to the small skin or skull incision but also emphasizes the minimal damage to brain tissue. In addition to being as effective and safe as the standard craniotomy, the supraorbital keyhole approach offers additional benefits such as fewer postoperative complications, faster recovery, and improved cosmetic outcomes (3, 9). A study conducted by Nohra Chalouhi et al. compared the safety and effectiveness of the supraorbital keyhole approach with the standard pterional approach for the treatment of ruptured anterior circulation aneurysms. The results showed that the supraorbital group had significantly shorter operation times (205 min) compared to the pterional approach group (256 min, *p* < 0.01). Although the postoperative complication rate was slightly higher in the supraorbital group compared to the pterional approach group (23.4% vs. 17.5%, *p* = 0.4), the proportion of patients with a good outcome (Glasgow Outcome Scale score of 4 or 5) at the 1-year follow-up was similar in both groups (76.6% vs. 75%, *p* = 0.8) (10). In a systematic review conducted by Wen-qiang Xin et al., multiple clinical reports on the treatment of intracranial aneurysms using the supraorbital keyhole approach and the pterional approach were analyzed. The review concluded that the supraorbital approach had shorter operation and hospitalization times and a lower risk of postoperative infection. Furthermore, the efficacy and safety of the supraorbital approach were comparable to those of the pterional approach (11). Zoe M. Robinow’s meta-analysis of the supraorbital approach for the treatment of intracranial aneurysms and tumors also supports the finding that the supraorbital approach is a safe and feasible minimally invasive surgery (12).

With the advancement of optical technology, endoscopes have addressed the limitations of traditional microscopes in terms of illumination. Endoscopes can provide deep illumination without creating shadows, leading to enhanced visualization of the surgical area and better differentiation of lesions and bleeding points (13). Additionally, the ability to view the interested area from different angles allows for comprehensive coverage, leading to improved lesion clearance, hemostasis capability, and reduced risk of postoperative rebleeding. In contrast, traditional microscopes require adjustments in angle to illuminate the surgical area, but there may still be unilluminated zones. Through Zoe M. Robinow’s meta-analysis, it was found that both the endoscopic group and the non-endoscopic group achieved high success rates in treating intracranial aneurysms and tumors using the supraorbital approach. Furthermore, the endoscopic group exhibited a lower complication rate in the treatment of tumor diseases (8.4 ± 3.4% vs. 20.2 ± 2.3%, *p* = 0.041) (12). In the management of hemorrhagic stroke, endoscopic surgery has demonstrated higher rates of hematoma clearance, lower complication and mortality rates, and better overall prognosis. In a study conducted by Yuqian Li et al., a comparison was made between endoscopic surgery, craniotomy, and stereotactic aspiration. The results indicated that endoscopic surgery exhibited superior safety and therapeutic effects, particularly in patients with a hematoma volume exceeding 60 mL and a Glasgow Coma Scale score of 4–8 points (14). Furthermore, endoscopic surgery is associated with less trauma, shorter operation times, faster recovery, and early mobilization of patients. These factors contribute to the reduction of complications such as pulmonary infection and thrombosis, as well as a shortened hospital stay.

Endoscope-assisted removal of traumatic frontal hematoma via the supraorbital approach has been rarely studied. Shuguang Zhang et al. conducted a study comparing the treatment of unilateral-dominant bilateral frontal contusion through the supraorbital approach under the microscope with the frontotemporal approach. The supraorbital approach group had 23 out of 26 patients with a hematoma clearance rate exceeding 90%, while the frontotemporal approach group had 35 out of 36 patients (*p* > 0.05). The supraorbital approach resulted in a shorter operation time (82.7 ± 13.73 vs. 132.4 ± 9.17 min, *p* < 0.05) and lower blood loss compared to the frontotemporal approach group (17.1 ± 4.55 vs. 67.6 ± 10.28 mL, *p* < 0.05). There were no significant differences in the incidence of postoperative rebleeding, intracranial infection, and GOS score after 6 months. This study confirmed the feasibility and safety of removing frontal hematoma using the supraorbital approach under the microscope (2). Additionally, Hyuk-Jin OH reported a study on the endoscopic supraorbital removal of frontal hematoma, where four patients underwent orbital resection. Among the 13 patients, ten achieved a hematoma clearance rate above 80%, with no significant postoperative change in hemoglobin levels (<1 g/dL), and most patients had good outcomes (15).

The supraorbital approach is associated with two common complications: frontal sensory disturbance following supraorbital nerve injury and intracranial infection after frontal sinus opening. An autopsy revealed that the angle between the lateral branch of the supraorbital nerve and the orbit ridge is 74 ± 3 degrees. There is no ramus within 10 mm after the supraorbital nerve exits the supraorbital hole/incisure (16). Thus, maintaining an incision outside the supraorbital hole/incisure of at least 5 mm can minimize supraorbital nerve damage. Additionally, careful use of electro excision and identification/protection of the supraorbital nerve can further reduce damage (12). Interestingly, although opening the frontal sinus theoretically increases the risk of intracranial infection and cerebrospinal fluid leakage, many studies do not show a higher infection rate when using the supraorbital approach, even when frontal sinus opening is involved. Conversely, some reports indicate that standard craniotomy has a higher infection rate, which may be attributed to the larger injury and longer operation time associated with this approach (11). Compared to the frontotemporal approach, the supraorbital approach’s smaller incision spares the frontal branch of the facial nerve, superficial temporal artery, and temporalis muscle from injury. By designing the incision at the eyebrow, minimizing muscle dissection, and separating along the muscle fiber without disrupting it, the cosmetic impact of the operation can be minimized, which is particularly important for some female patients (3, 17).

In contrast to procedures like aneurysm clipping and tumor resection, which involve opening the subarachnoid space and releasing cerebrospinal fluid to create more space, the frontal hematoma is always accompanied by cerebral edema, and the space-occupying effect of hematoma will further compress the operating space. Based on our experience, we recommend gradually removing the hematoma along the channel after entering the hematoma cavity. However, if the hematoma is in an area with limited visibility or difficult instrument access, it may not be necessary to remove it entirely, because not all hematomas and brain contusions require removal, as doing so may lead to difficult hemostasis and potential new bleeding. Most hematomas can be easily removed, but larger hematomas should be removed in smaller pieces. The size of the bone flap is also crucial, with a minimum requirement of 1.5–2.0 cm to facilitate the use of microsurgical instruments. Additionally, we recommend considering the endoscopic supraorbital approach in established and well-structured neurotrauma service units (18). This is due to the technical complexity and demanding nature of both the supraorbital and endoscopic approaches, which have steep learning curves (19). Therefore, it is advisable to have experienced neurosurgeons perform these procedures to enhance surgical safety and minimize complications. Furthermore, postoperative monitoring and management are critical. Unlike the frontotemporal approach, non-invasive monitoring techniques are typically employed after the supraorbital approach. It is essential to utilize reliable and comprehensive non-invasive multimodality neuromonitoring, such as near-infrared spectroscopy and transcranial doppler ultrasonography, to promptly detect intracranial complications or secondary injuries (20, 21).

The study has several limitations, including a small sample size, retrospective analysis, and being conducted at a single center. Additionally, the severity and complexity of trauma may also impact the outcome, in addition to surgical options. The frontotemporal approach group in the study included some cases with serious conditions, which could potentially undermine the evaluation of the treatment effect of the frontotemporal approach. This may lead to results that are more favorable toward the supraorbital approach. Therefore, when the cases in both groups are totally comparable, it remains unclear whether the results are still reliable and this requires confirmation through high-quality research. It is important to note that the objective of our study was to investigate the feasibility and safety of the endoscopic supraorbital approach for treating frontal hematoma, and to demonstrate that it can serve as an alternative surgical method for certain patients, rather than replacing the traditional frontotemporal approach for all patients.

## Conclusion

The endoscopic supraorbital approach appears to be a viable, safe, and effective surgical approach for treating traumatic frontal hematoma. Compared to the frontotemporal approach, the supraorbital approach has shorter operation times and less blood loss. There were no significant differences in hematoma clearance rate, postoperative complications, and outcomes between the two groups. However, further confirmation through high-quality randomized controlled studies is necessary.

## Data availability statement

The original contributions presented in the study are included in the article/supplementary material, further inquiries can be directed to the corresponding author.

## Ethics statement

Written informed consent was obtained from the individual(s) for the publication of any potentially identifiable images or data included in this article.

## Author contributions

QY and XT: conception and design. QY, MC, WX, and WZ: acquisition and analysis of data. YW and YL: drafting the article. PC and XT: critically revising the article. All authors contributed to the article, reviewed, and approved the submitted version.

## Conflict of interest

The authors declare that the research was conducted in the absence of any commercial or financial relationships that could be construed as a potential conflict of interest.

## Publisher’s note

All claims expressed in this article are solely those of the authors and do not necessarily represent those of their affiliated organizations, or those of the publisher, the editors and the reviewers. Any product that may be evaluated in this article, or claim that may be made by its manufacturer, is not guaranteed or endorsed by the publisher.

## Abbreviations

CT: Computed tomography
GCS: Glasgow Coma Scale
GOS: Glasgow Outcome Scale
